# The discordance between clinical and radiographic knee osteoarthritis: A systematic search and summary of the literature

**DOI:** 10.1186/1471-2474-9-116

**Published:** 2008-09-02

**Authors:** John Bedson, Peter R Croft

**Affiliations:** 1 Arthritis Research Campaign National Primary Care Centre, Keele University, Staffordshire, ST5 5BG, United Kingdom

## Abstract

**Background:**

Studies have suggested that the symptoms of knee osteoarthritis (OA) are rather weakly associated with radiographic findings and vice versa. Our objectives were to identify estimates of the prevalence of radiographic knee OA in adults with knee pain and of knee pain in adults with radiographic knee OA, and determine if the definitions of x ray osteoarthritis and symptoms, and variation in demographic factors influence these estimates.

**Methods:**

A systematic literature search identifying population studies which combined x rays, diagnosis, clinical signs and symptoms in knee OA. Estimates of the prevalence of radiographic OA in people with knee pain were determined and vice versa. In addition the effects of influencing factors were scrutinised.

**Results:**

The proportion of those with knee pain found to have radiographic osteoarthritis ranged from 15–76%, and in those with radiographic knee OA the proportion with pain ranged from 15% – 81%. Considerable variation occurred with x ray view, pain definition, OA grading and demographic factors

**Conclusion:**

Knee pain is an imprecise marker of radiographic knee osteoarthritis but this depends on the extent of radiographic views used. Radiographic knee osteoarthritis is likewise an imprecise guide to the likelihood that knee pain or disability will be present. Both associations are affected by the definition of pain used and the nature of the study group. The results of knee x rays should not be used in isolation when assessing individual patients with knee pain.

## Background

There is a widespread belief that there is a high discordance between clinical and radiographic knee osteoarthritis (OA) [1-3]. However, this belief contrasts with the assumption that osteoarthritis is the commonest knee pathology in older people and the commonest reason for knee pain and disability in this age-group, and that radiographs appropriately identify moderate and severe osteoarthritis. Therapeutic options such as surgery for knee pain are considered in the presence of radiographic abnormalities [4]. In previous work we have shown that the presence of radiographic knee osteoarthritis can influence the decision of general practitioners in their management strategies, particularly leading to increased levels of referral to secondary care [5]. It is therefore important to understand the apparent lack of association between pain and knee x rays, particularly if the best clinical choices for patients are to be made and the basis for these choices clearly established. There has been no previous review of all studies which have investigated the association between pain and x rays at the knee. This paper seeks to fill that gap.

The features revealed by a knee x ray serve its main purpose as a diagnostic tool. However, in common with other diagnostic tests, the x ray also supports several other potential applications. These include estimating prognosis, guiding treatment, post therapeutic evaluation, giving reassurance to patient or physician and helping to preserve the doctor-patient relationship [6]. One study has indicated that GPs use x rays as a part of their management strategy because they perceive them as being helpful in making management decisions, such as avoiding unnecessary referrals to specialists, and in providing a useful aid to discussing management with patients [7]. However this means that the relationship between x ray findings and clinical complaints is crucial to understand if decisions based on x ray findings are going to appropriately influence what the patient considers important, namely reducing pain and disability.

As previously stated, population studies have suggested that the 'fit' between x rays and symptoms at the knee is not perfect. This paper describes a systematic search of the literature to identify the extent of these discrepancies and the possible reasons why they might arise. In general there are two possible reasons for the discrepancy. Firstly the way in which radiographic osteoarthritis is defined will affect the number of cases classed as having radiographic disease or not, and therefore the prevalence of radiographic OA disease. In the knee for example the joint has three compartments. If the only x rays considered are the antero-posterior view, then only osteoarthritis in the medial and lateral compartments would be identified and up to 24% of patients with radiographic knee OA would be missed by not visualising the patello-femoral joint [8]. Secondly clinical symptoms and signs may arise from sources other than the contents of the knee joint or the underlying subchondral bone, and so the ways in which the clinical syndrome of osteoarthritis is defined will influence the extent to which it is linked with osteoarthritis defined on a knee X-ray.

The first objective of this systematic review was to identify studies which provide an estimate of the prevalence of radiographic knee OA in older people with knee pain. The second objective was to determine what influences this prevalence and therefore might be a source of error or variation in the observed associations between x rays and symptoms: namely the definition of x ray osteoarthritis, the definition of symptoms, and the effect of demographic factors such as age and ethnicity.

## Methods

The strategy and keywords for the search are given in appendix I. The first step of the strategy was to identify papers that included reference to knee osteoarthritis in the various forms by which it can be referred to in the literature, and all papers relating to diagnosis and clinical signs in knee OA. The next step was to filter these papers to extract those which included radiographic investigation and to limit them to extract those which concerned population-based observational studies and not intervention studies. Other exclusions at this stage were papers about arthritic conditions other than osteoarthritis, modes of investigation other than x rays, such as MRI, and papers in non-English languages. This search strategy therefore identified papers which combined x rays, diagnosis, clinical signs and symptoms in knee osteoarthritis in population studies.

Two databases were used, EMBASE and Medline. The initial search identified 134 papers. These were then examined by title to include papers in the review which specifically related to knee pain, knee symptoms, knee x rays or the prevalence of any these factors. This limited the papers to 60. The abstracts for these papers were then assessed to determine if the paper contained at least one x-ray view of the knee and mentioned at least one knee related symptom. Applying these criteria limited the review to 20 papers.

### Analysis

The first analysis of the results considers papers from which estimates of the prevalence of radiographic OA in people with knee pain can be derived. The second analysis considers those papers from which the prevalence of knee pain can be derived in populations of people defined as having radiographic knee OA.

The analysis considered three factors that might potentially influence these associations. There are a range of factors that might explain or influence discordance between radiographs and symptoms. We chose three of these – age, gender, ethnicity – to test the hypothesis that discordance might vary between population sub-types.

#### 1. The nature and extent of radiographic views

One potential factor that might lead to apparent lack of association between x rays and symptoms is that in the studies conducted, there were insufficient numbers of x rays – for example of persons with very severe pain – to provide the power to detect strong associations overall. To overcome this, we decided to include all radiographic views of the knees used in these papers. The associations of clinical features with different radiographic views were either drawn directly from the results in the paper or were calculated if the raw data allowed. Sensitivities and specificities for the varying views and grades in relation to symptoms were examined. Where appropriate, odds ratios were examined for the relationships.

#### 2. The definition of symptoms

A second factor that might be related to the lack of association was the pain itself. Pain comes in many forms, and is an individual experience. However research has to attempt to standardise the approach to this experience so that it can be measured. We therefore explored all levels and definitions of pain. Papers that examined symptoms were included and their definition of pain identified and catalogued. The prevalence of radiographic knee OA in relation to these definitions was identified and used to estimate the proportions of knee pain sufferers who have radiographic OA. Where appropriate, odds ratios were examined for the associations between pain and radiographic OA. Also included were papers which used the Western Ontario and McMaster Universities Arthritis Index (WOMAC) as an alternative methodology for studying the relationship between symptoms and radiographic knee OA [9]. The WOMAC has several sections which cover knee pain, stiffness and function. As an example, the pain-specific subset for the knee assesses the severity of pain during five activities including walking on a flat surface, going up or down stairs, at night while in bed, sitting or lying and standing upright. Each category is assigned a numerical score of 1 to 5, corresponding to the severity of pain (none, mild, moderate, severe and extreme). Such a scale allows for a more detailed analysis of the relationship between symptoms and x rays.

#### 3. The nature of the study group

A third important factor which could influence the association within these studies is the population under scrutiny. If this lack of association is real, then it should be true for all groups equally. We have tested this by selecting three population characteristics – age, gender and ethnicity – and investigated whether they should be taken into account in estimating discordance between pain and radiographic knee OA. Papers were included which examined the differences in prevalence of radiographic knee OA and knee symptoms according to age, gender and ethnicity as examples of external factors that might influence the nature and extent of the association between symptoms and radiographic features. Prevalence estimates for knee pain and radiographic knee OA according to age bands and ethnic groups were collated, and odds ratios calculated for the associations.

## Results

### Section 1: The prevalence of radiographic osteoarthritis in people with knee pain

Table 1 summarises the estimates of prevalence from the studies reviewed of persons with knee pain found to have x ray abnormalities consistent with radiographic knee OA. Knee pain was the most frequent marker symptom and has been used to construct this main table. However other symptoms were reported in the different studies, but definitions varied and could not be used to compare the study results. Pain, by contrast, featured in all the studies and therefore provided a common factor to which to relate estimates of the frequency of radiographic knee osteoarthritis. The figures are shown stratified by age, with the youngest group first and older age groups further down the table. The different radiographic views used are highlighted, as are the definitions used to classify an abnormal radiograph as showing osteoarthritis.

**Table 1:** Proportion (%) of patients who have radiographic osteoarthritis in specified age-groups of populations with knee pain.

Study	Age Group	Radiographic View	Proportion (%)	OA Definition	Population
Petersson [19]	31–54	A/Pwb	15	Ahlb ≥ 1	All
				K&L 2+	
Lachance [13]	40–53	A/P	15	K&L 2+	CA
			40		AA
Hart [18]	45–65	A/Pwb	19	K&L 2+	All
Hannan [25]	51–74	A/P	15	Def Ost	All
Lanyon[8]	40–80	A/Pwb + S/L	Grade 1+ 63%	Altman	All
			Grade 2+ 30%	≥ Grade I	
			Grade 3 12%	Ost [32]	
Claessens [3]	> 45	A/Pwb	36	K&L 2+	All
Cicuttini [11]	> 45	Lateral Flexed wb	30	Def	Female
		S/L	53	Ost	Female
Cicuttini [26]	> 45	A/Pwb	37	Ost	Female
		Lateral	37	JSN	Female
		S/L	51	or both	Female
Odding [14]	> 55	A/Pwb	39	K&L 2+	All
McAlindon [17]	> 55	A/Pwb + Lateral	76	K&L 2+	All
Brandt [24]	> 65	A/Pwb + Lateral	49	K&L 2+	All
Lethbridge [10]	19–92	A/P	KL 2+ 53%	K&L 2+	All
			KL 3+ 22%		
			KL 4 2%		
Williams [21]	51–80	A/Pwb + Lat Flexed	43	K&L 2+	All

A/P – antero-posterior

wb – weight bearing

S/L – skyline view

Lat – lateral

CA – Caucasian

AA – African American

All – whole population

K&L – Kellgren & Lawrence Knee OA Grading Scale [33]

Ahlb – Ahlbäck Knee OA Grading Scale [34]

Ost – Osteophytes

Def Ost – Definite Osteophytes

JSN – Joint Space Narrowing

The proportion of those with knee pain found to have radiographic osteoarthritis ranged from 15–76%. One study which encompassed a wide age range (19 – 92 years) found that 53% of current knee pain sufferers had radiographic knee osteoarthritis [10].

#### The x ray view

Table 1 indicates the various x ray views employed in the individual studies. The antero-posterior (A/P) view was employed in all except one and in most of the studies the weight-bearing view was used. Additional views of the joint were employed in several of the studies reviewed including lateral (mediolateral), lateral flexed and skyline views (inferosuperior). Which views are used in the varying studies appears to have some impact upon the relationship of pain to radiographic knee OA. Claessens uses only the A/P weight bearing view and identifies 36% of patients with knee pain as having radiographic knee OA [3]. Lanyon uses the A/P weight bearing in conjunction with the lateral and identifies 53% [8]. Cittucini meanwhile observes that 53% of patients with knee pain have radiographic knee OA when using the skyline in isolation [11]. Knee studies that include x rays of the patello-femoral joint (PFJ), improve the sensitivity with which symptoms such as pain can identify radiographic knee OA to a potential 51–67% [8,12,13]. Excluding this view drops the sensitivity to 24–38% [3,8,14]. It appears that discrepancy between knee symptoms such as pain and radiographic knee OA is due in part to not employing x rays of all three compartments of the knee. However this does not explain all the discrepancy, since even when all compartments are x rayed the highest proportion of patients with pain who have radiographic knee OA is 76% [15]. A recent paper from our unit not included in the review suggests that systematically searching all three X-ray views of the knee for evidence of OA in persons over 50 years with knee pain identifies OA in 70% [16].

#### Grading the x ray

Grading an x ray entails defining the level of abnormality found in an x ray considered to represent knee osteoarthritis. Increasingly abnormal features may be added to this base level to define increasing severity. Table 1 shows the x ray knee OA definitions used in the studies. Common to all the studies was the use of osteophytes at some point in the 'baseline' definition. Ciccutini and Lanyon both used Grade 1 osteophytes (minute) [8,11], all the others used grade 2 (definite) or 'definite osteophytes' as the main defining feature. The use of grade 1 versus grade 2 appears to make little difference between studies. However, the association of knee pain and x ray grade was investigated by McAlindon who found a limited but positive correlation between knee pain and x ray grade (Pearson's correlation coefficient = 0.43) [17].

Classically the Kellgren and Lawrence grading scale has rated joint space narrowing as grade 3 with osteophytes occurring at grade 1 or more. Cicuttini found that knee pain was significantly associated with osteophytes in all x ray views but not with joint space narrowing. As an example in the A/P view the odds ratio for the association of osteophytes and ever having had knee pain (episodes lasting more than 15 days) was stronger and significant (OR 5.0;95% CI 3.01,11.33) when compared with the odds ratio for pain and joint space narrowing alone (OR 2.13; 95% CI 0.78,5.87) [11]. The association of knee pain with osteophytes was also examined by Lanyon who estimated that of knee pain positive subjects, 12% were K/L osteophyte grade 3, whilst 30% were grade 2 or above. When the lowest grade of osteophyte was included (grade 1), 63 % of knee pain sufferers were classified as having radiographic osteoarthritis [8]. Lethbridge also found increased levels of radiographic OA when using more inclusive grades with 53% of current knee pain sufferers having K/L grade 2 or more, but only 22 % of those with pain had K/L grade 3 and above [10]. In addition, Hart, analysed the sensitivity and specificity for the association of knee joint pain with K/L grade 1 or more and compared this to grade 2 or more and found no difference (23% sensitivity, 88% specificity) [18].

#### Defining knee symptoms

Table 2 demonstrates how the proportion who have radiographic knee OA varies with the definition of knee pain. There are 10 different definitions used. These vary considerably, from 'ever having an episode of pain lasting 15 days or more' [11] to 'knee pain during the past month' [14]. There is corresponding variation in the prevalence of radiographic knee OA depending upon the definition of pain used. Where the definition is one that involves recalled pain over a specific period, such as in Petersson's study, the prevalence is lower (15%) [19] than for pain "ever", as in Ciccutini's study (37%) [11] or recent pain as in Odding's (39%) [14]. Even when the same question is used for different studies, a wide variation in prevalence is evident [8,10,15,17]. As table 2 details, Felson and colleagues used similar definitions of pain to these [20], but only part of them were used to define a patient as knee pain positive. Felson's results indicated only 16% of patients with knee pain had radiographic knee OA, compared with a range of 30–76% in the other studies [8,10,15,17].

**Table 2:** Proportion (%) of people with radiographic knee OA in populations with knee pain according to the definition of knee pain.

Study	% Radiographic OA in those with Knee pain	Definition of knee pain positive subjects
Hannan [25]	15	Pain, swelling, morning stiffness in or around the knee on most days for one month

		Positive response to both parts required:
Lanyon [8]	30	(A) Have you ever had pain in or around the knee on most days for one month?
McAlindon [15]	53	
McAlindon [17]	76	
Lethbridge [10]	53	(B) If so, have you experienced pain in the last year?
Felson [20] (Part A only)	16	

Cicuttinni [11]	37	Ever having an episode of knee pain
Cicuttinni [26]	30	Ever having an episode of knee pain lasting more than 15 days

Peterson [19]	15	Pain in your knees practically daily for the last 3 months
Lachance [13]	15 (CA) 40 (AA)	Any joint pain in their knees during the last during the last month
Hart [18]	19	Pain, stiffness and swelling lasting more than a month
Odding [14]	39	Knee pain during the past month
Jordan [35]	N/A	Knee pain on most days
Davis [27]	N/A	Knee pain on most days lasting for one month in the past year

Williams [21]	N/A	
Brandt [24]	N/A	
Ang [23]	N/A	

CA – Caucasian, AA – African American, N/A – not applicable.

Other studies have employed the WOMAC to examine knee symptoms but in different ways as shown in table 3[21-24]. A direct comparison is not possible due to the variation in the definition of a knee pain positive patient, but despite this variation, there is overall no significant difference in WOMAC pain score between knee pain positive patients with radiographic knee OA and those without it.

**Table 3:** Comparison of WOMAC scores between studies employing varying definitons of knee pain positive patients.

Study	Pain +ve WOMAC definition	WOMAC score in those with knee pain but no radiographic knee OA compared to those with radiographic knee OA
Brandt [24]	Greater than moderate (> 3) for any of the five categories on more than half the days in the month preceding evaluation	No significant difference
Williams [21]	Currently had mild pain or greater (> 0).	No significant difference
Ang [23]	Current or past pain, WOMAC transposed to a scale of 0 – 100	No significant difference

#### The nature of the study group

Younger age groups with knee pain have a lower prevalence of radiographic knee osteoarthritis than older persons [13,19]. Restricting analysis to persons aged between 40 and 80, the proportion of knee pain sufferers with radiographic osteoarthritis is 19–30% [1,8,25]. For all those aged over 45 this rises to 36–50% [3,11,21,24,26], and over 55 the range is 40–76% [14,15,17]. Several studies support the age-related nature of the changes found in radiographic knee osteoarthritis in those with knee pain [3,10,12,25]. As an example, in Hannan's study, the prevalence was 2 % in those aged 25 – 40, clearly less than the 21% estimate among those aged 51–74 [25]. In only one study was this trend not evident [27].

Two studies investigated the prevalence of radiographic knee osteoarthritis in both Caucasian and African American subjects with knee pain [13,23]. Lachance identified higher levels of radiographic knee osteoarthritis in African American (AA) than Caucasian (CA) women with knee pain (40% vs. 15%) [13]. The overall level of radiographic osteoarthritis was higher for the African Americans (23.2%) compared to the Caucasians (8.5%). The age range in this study was 40–53. In Ang's study of men and women over 50 (average 65 years) [23], the overall prevalence of knee osteoarthritis was similar for both ethnic groups (AA 39.4%; CA 38.7%), but the severity of the K/L grading was significantly higher in the presence of larger osteophytes in African Americans compared to Caucasians. With respect to the sensitivity with which pain could predict radiographic knee osteoarthritis, Lachance demonstrated that this was higher for African American women (51%) compared to Caucasian women (35%), but the specificity for Caucasian women was higher (CA 85%; AA 77%) [13].

### Section 2: The prevalence of knee pain and clinical osteoarthritis in people with radiographic osteoarthritis

Table 4 summarises the studies that give estimates of the prevalence of knee pain for specific age groups from a population found to have abnormal knee radiographs. The different radiographic views are highlighted. There is a large variation in the proportion of those with radiographic knee OA who experienced pain, ranging from 15% – 81%.

**Table 4:** Proportion (%) of patients experiencing knee pain in specified age-groups of populations with radiographic osteoarthritis.

Study	Age Group	Radiographic View	Proportion (%)	OA Definition	Population
Lachance [13]	40–53	A/Pwb	35	K&L 2+	CA
			50		AA
Hart [18]	45–65	A/Pwb	56	K&L 2+	All
Davis [27]	45–75	A/Pwb	41(KL2)	K&L I+	All
			59(KL3)		All
Hannan [25]	51–74	A/P	47	Def Ost	All
Claessens [3]	> 45	A/Pwb	24	K&L 2+	All
Cicuttini [11]	> 45	Lateral Flexed wb	16	Def Ost	Female
		S/L	26		Female
Cicuttini [26]	> 45	A/Pwb	20	Ost	Female
		Lateral	15	JSN	Female
		S/L	23	or both	Female
Odding [14]	> 55	A/Pwb	30(KL2)	K&L 2+	All
			59(KL3)		
Felson [20]	> 63	A/Pwb	40	K&L2+	All
				or JSN	
Brandt [24]	> 65	A/Pwb + Lateral	22	K&L2+	All
Lethbridge [10]	19–92	A/P	30(KL2)	K&L2+	All
			64(KL3)		
Williams [21]	51–80	A/P Lat Flexed	79	K&L2+	All
Lanyon [8]	40–80	A/Pwb + S/L	81	Altman	All
				≥ Grade I Ost [32]	

A/P – antero-posterior

wb – weight bearing

S/L – skyline

Lat – lateral

CA – Caucasian

AA – African American

KL – Kellgren Lawrence grade

All – whole population

K&L – Kellgren & Lawrence Knee OA Grading Scale [33]

Ost – Osteophytes

Def Ost – Definite Osteophytes

JSN – Joint Space Narrowing

#### The x ray view

Considering those studies where an A/P view alone is used, between 24 – 56% of patients with radiographic knee osteoarthritis experience pain [1,3,10,13,14,20,25,27,28]. If lateral views alone are considered then 15% of patients with radiographic OA on this view have pain [11]. Adding a lateral or skyline to the A/P view increases the prevalence of pain in those with radiographic OA to 80% [8,21]. Cicuttini's study found that abnormalities in the skyline view were nearly twice as likely to predict knee pain as a lateral view, and were also superior to the A/P view in doing this [11]. Including views of the patello-femoral joint improved the sensitivity of predicting knee pain from 38% to 62% in one study [8], and by 10% to 50% in another [20], but with corresponding reductions in specificity.

With respect to disability, Odding found that abnormalities in the knee x ray were weak predictors of locomotor disability in women and not at all in men [14]. Davis similarly found no association with disability, even for severe radiographic knee osteoarthritis when controlling for other variables such as age, sex and BMI [27]. McAlindon identified ageing, knee pain and quadriceps weakness as three important factors associated with disability but there was no association with radiographic knee osteoarthritis [17].

#### Grading the x ray

Higher grade of osteoarthritis (K/L 3 or more) is a stronger predictor of the presence of pain than lower grades (K/L 2 or less) [8,10,11,14,20,27]. Table 4 illustrates three studies in which this is apparent, for example Odding found that knee pain was nearly twice as likely for K/L grade 3 as for lower grades [14]. Felson's study of various definitions for knee osteoarthritis examined the use of different radiographic features and their association with the characteristics of clinical osteoarthritis such as pain. The highest sensitivity found was with any grade one osteophyte (82.5%), but the specificity was low (23.3%). On the other hand, joint space narrowing (K/L grade 3) had a low sensitivity (38.3%), but high specificity (82.9%) [20]. Cicuttini describes higher grades of osteophytes as significantly associated with knee pain in the skyline view, but not in the lateral view [26].

#### Defining pain

Table 5 examines the proportion of people with varying definitions of knee pain in populations with radiographic knee OA. Definitions which examined 'current' pain found prevalence rates of this symptom in radiographic-positive groups that varied from 59 – 81% [8,10,14,27]; lower prevalence estimates were found in studies of pain 'ever', varying from 20 – 59% [10,18,20,25,26]. Even within studies variations existed between 'ever' and 'current' pain. Lethbridge (see table 5) estimated that the prevalence of pain at some time in or around the knee for one month among persons with radiographic OA was 53%, but for the same group, if this was limited to experiencing the pain in the last year, this increased to 64%. Cicuttini describes how osteophytes on any view were better predictors of pain in the knee during the last year than pain in the last month or 'ever' [11]. This provides limited evidence that the type of recalled pain might be linked with radiographic pain.

**Table 5:** Proportion of knee pain positive patients with radiographic knee OA (A/P views) according to the definition of knee pain.

Study	% Knee pain positive in those with Radiographic OA	Definition of knee pain positive subjects
Hannan [25]	47	Pain, swelling, morning stiffness in or around the knee on most days for one month

		Positive response to both parts required:
**Parts A & B**		(A) Have you ever had pain in or around the knee on most days for one month?
Lanyon [8]	81	
Lethbridge [10]	64	
**Part A only**		(B) If so, have you experienced pain in the last year?
Felson [20]	40	
Lethbridge [10]	53	

Cicuttinni [26]	20	Ever having an episode of knee pain lasting more than 15 days
Lachance [13]	35 (CA) 50 (AA)	Any joint pain in their knees during the last during the last month
Hart [18]	56	Pain, stiffness and swelling lasting more than a month
Odding [14]	59	Knee pain during the past month
Davis [27]	59	Knee pain on most days lasting for one month in the past year

CA – Caucasian, AA – African American, N/A – not applicable.

#### Nature of the study group

There appears to be no consistent relationship between age and prevalence of pain in populations with radiographic knee OA. Table 4 shows the prevalence of knee pain among patients with radiographic knee osteoarthritis in a specified age-group of the population. Williams and Lanyon looked at older age groups and found that about 80% of patients had knee pain [8,21]. Lethbridge considered a much wider age range from 19 – 92 and found lower proportions with pain for both K/L grade 2 (30%) and grade 3 (64%) [10]. However the findings of those studies looking at patients in their 40's and over [3,11,13,14,18,25-27], were not markedly different to those looking at patients aged in their 60's and over [20,24].

Two studies considered differences between African Americans and American Caucasians from similar geographic locations [13,23]. Table two shows that American Caucasians with radiographic knee OA were less likely to experience pain compared to African Americans (35% vs. 50%) [13], whereas Ang found no ethnic differences in the WOMAC pain and function score for any given level of radiographic knee osteoarthritis [23].

## Discussion and conclusion

This examination of the literature has revealed a wide variation in the degree to which knee pain relates to radiographic knee osteoarthritis and vice versa. We postulated that there might be three particular reasons as to why discordance between x rays and symptoms might arise, from which we can now draw three main conclusions.

Firstly there may be insufficient x ray numbers or views used to estimate the association. The studies show that the prevalence of radiographic knee OA will be underestimated in persons with knee pain in studies that do not obtain all potential x ray views of the knee. This is supported by the finding that knee studies including x rays of the patello-femoral joint (PFJ), improve the sensitivity with which symptoms such as pain can identify radiographic knee OA [8,12,13]. By adding a lateral or skyline to the A/P view, overall prevalence of radiographic knee OA in pain positive persons increases to 80% [8,21]. A recent paper from our group, subsequent to this review, has confirmed this conclusion by showing directly that the prevalence of overall radiographic OA of the knee increases with the number of radiographic views in a population with knee pain [16]. However, much discordance remains between pain and x ray findings, and no combination of views reaches a point where patients with knee pain invariably have radiographic knee OA. This is also true for studies examining the prevalence of pain in populations with radiographic knee OA. There is a great deal of discordance evident amongst these studies as highlighted in Table 5. Overall these studies support the conclusion that the lack of association between radiographic knee OA and pain is to some extent a real one.

**Figure 1:**
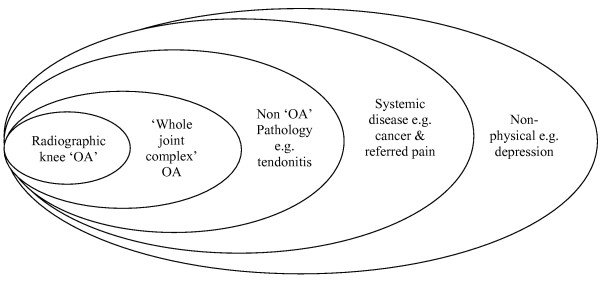
Sources of chronic knee pain.

Secondly, the way pain is defined (e.g. whether disability is included or not) and the grading of radiographic severity, have important influences upon estimates of association between knee pain and radiographic OA and vice versa. Table 2 and table 4 examined this relationship with respect to pain definition and demonstrate the wide variation in pain definitions used, and the correspondingly wide variations in the associations between knee pain and x ray findings. It seems likely that the often observed discrepancy between pain and radiographic knee OA has something to do with this variation in definition of pain, and that, if similar methods of pain definition were used, some consistency in the level of discrepancy might emerge. However, the variation between studies is quite marked, so one cannot be wholly convinced of the idea that using one standard uniform definition will lead to x rays and pain becoming more concordant. Other reasons might play their part here. Figure 1 shows the sources of chronic knee pain in the older person that as a whole make up the knee 'pain picture' we encounter in general practice. Pain in the knee is more than just the result of the pathological changes reflected in the x ray. Other factors may account for knee pain which will not be evident on the knee x ray. Figure 1 clearly shows this, indicating that the pain may be the result of other bone problems, not visible on an x ray such as oedema, or non-OA conditions such as ligament injury or tendonitis. Indeed, some chronic knee pain might be more strongly linked to issues of cognitive or emotional state such as depression rather than local pathology at the knee joint. Of course, all these things can coexist at the same time, making up multiple layers of causality of knee pain

The complementary problem concerns the variation in definitions of radiographic OA in any particular view. Some would argue that an isolated osteophyte is not osteoarthritis, although whether the mildest form of osteophyte is included in the definition of OA or not seems to make little difference to the association with pain. However what is clearer from the papers we reviewed is that, with respect to the x ray grade, at the severe end of the spectrum there is a closer association of pain and x rays as shown in table 1 and table 4, but milder disease is more common and the discordance evident at lower levels of K/L grade is important to consider in studies of knee pain and OA. The way the x ray is taken is also important. Between studies the radiographic technique employed may have differed. This will have encompassed whole protocols which might involve the position of the knee (semi-flexed or straight knee). In addition reading the radiographs requires consistency. The studies described go to great lengths to attain intra-study consistency, but we are unable to comment on inter-study consistency and this must be taken into account when evaluating the findings between studies.

Thirdly, the nature of the study population is important since variations in the association of knee pain and radiographic knee OA may be influenced by characteristics of the population sampled. Younger age groups with knee pain are less likely to have radiographic knee OA (table 1), and there is also some variation with age in the proportion of persons with radiographic knee OA and one study suggests that younger patients with radiographic knee OA are less likely to be symptomatic [10]. Ethnicity also has some influence over the relationship [13,23]. Study populations are of course more diverse than in age, gender and ethnicity alone, and it may be that other characteristics than these may both influence the link between x rays and pain, and vary between the populations studied. We did not investigate the effect of other characteristics in this study.

The major issue for future research is that commitment to more uniformity and standardisation in definitions is needed to allow comparability between studies, and to remove variability between studies as a factor obscuring accurate estimates of the 'true' association between x rays and symptoms at the knee. This would almost certainly involve x raying multiple views of the knee, in a standardised way using consistent protocols across research groups. Pain analysis needs to be similarly standardised, and as recently used in one paper [29], the WOMAC scale allows detailed analysis of pain and dysfunction. Pain grading is essential and might be achieved through using the von Korff Chronic Pain Grade to allow combined measurement of pain and disability severity [30]. Finally using a sampling frame that identified people with a wide range of severity and duration of knee pain, and unselected for their use of healthcare, would deliver a population that truly would be free of selection bias and comparable across study groups.

We conclude, inevitably, that knee pain is an imprecise marker of radiographic knee osteoarthritis, even in older age groups, but the extent of this imprecision depends heavily on the extent of radiographic views of the joint obtained. Radiographic knee osteoarthritis is likewise an imprecise guide to the likelihood that knee pain or disability will be present, although the more severe the radiographic osteoarthritis, the more likely there are to be accompanying symptoms. Both associations are affected by the definition of pain used and the nature of the study group. The experience of pain is multi-factorial in its origin, and factors such as patient depression play an important part in its manifestation, and this is as true of osteoarthritis and joint pain in older people as it is for pain of uncertain pathology in younger people [31]. Using x rays as a means for investigating knee pain, particularly in older people, requires these other factors to be taken into consideration, and the results of knee radiographs should not be used in isolation when assessing individual patients with knee pain.

## Competing interests

The authors declare that they have no competing interests.

## Authors' contributions

JB and PC conceived the study. JB designed and conducted the analysis. All authors contributed to the interpretation and writing of the paper, with prime responsibility taken by JB.

## Appendix – Search protocol for the systematic search and summary of the literature relating to radiographic knee osteoarthritis and knee pain

Please see Table 6

**Table 6:** Search protocol for the systematic search and summary of the literature relating to radiographic knee osteoarthritis and knee pain

1.	SEARCH:	KNEE$.TI,AB,SH,DE.
2.	SEARCH:	PATELL$.TI,AB,SH,DE.
4.	SEARCH:	(KNEE ADJ JOINT).TI,AB,SH,DE.
5.	SEARCH:	(GENU ADJ VALGUS).TI,AB,SH,DE.
6.	SEARCH:	(GENU ADJ VARUS).TI,AB,SH,DE.
7.	SEARCH:	1 OR 2 OR 3 OR 4 OR 5 OR 6
8.	SEARCH:	OSTEOARTHR$.TI,AB,SH,DE.
9.	SEARCH:	OA.TI,AB,SH,DE.
10.	SEARCH:	gonarthrosis.TI,AB,SH,DE.
11.	SEARCH:	(DEGENERATIVE ADJ JOINT ADJ DISEASE).TI,AB,SH,DE.
12.	SEARCH:	8 OR 9 OR 10 OR 11
13.	SEARCH:	DIAGNOS$.TI,AB,SH,DE.
14.	SEARCH:	GNOSIS.TI,AB,SH,DE.
15.	SEARCH:	PROGNOS$.TI,AB,SH,DE.
16.	SEARCH:	13 OR 14 OR 14 OR 15
17.	SEARCH:	(X ADJ RAY).TI,AB,SH,DE.
18.	SEARCH:	RADIOGRAPHIC$.TI,AB,SH,DE.
19.	SEARCH:	RADIOLOGICAL$.TI,AB,SH,DE.
20.	SEARCH:	RADIOLOGIST$.TI,AB,SH,DE.
21.	SEARCH:	17 OR 18 OR 19 OR 20
22.	SEARCH:	7 AND 12 AND 16 AND 21
23.	SEARCH:	MRI.TI,AB,SH,DE.
24.	SEARCH:	CT.TI,AB,SH,DE.
25.	SEARCH:	23 OR 24
26.	SEARCH:	22 NOT 25
27.	SEARCH:	ARTHROPLASTY.TI,AB,SH,DE.
28.	SEARCH:	(KNEE ADJ REPLACEMENT).TI,AB,SH,DE.
29.	SEARCH:	(KNEE ADJ SURGERY).TI,AB,SH,DE.
30.	SEARCH:	ARTHROSCOP$.TI,AB,SH,DE.
31.	SEARCH:	27 OR 28 OR 29 OR 30
32.	SEARCH:	26 NOT 31
33.	SEARCH:	gout$.TI,AB,SH,DE.
34.	SEARCH:	rheumatoid$.TI,AB,SH,DE.
35.	SEARCH:	pseudogout$.TI,AB,SH,DE.
36.	SEARCH:	33 OR 34 OR 35
37.	SEARCH:	32 NOT 36

## Pre-publication history

The pre-publication history for this paper can be accessed here:


